# Outcomes of locally advanced gastric and gastroesophageal adenocarcinoma cancers treated with neoadjuvant FLOT in a tertiary care hospital in Pakistan

**DOI:** 10.3332/ecancer.2024.1705

**Published:** 2024-05-21

**Authors:** Tasneem Dawood, Yasmin Abdul Rashid, Saqib Raza Khan, Adnan Abdul Jabbar, Muhammad Nauman Zahir, Munira Shabbir Moosajee

**Affiliations:** 1 Department of Medical Oncology, Aga Khan University Hospital, Karachi 74800, Pakistan; 2 Department of Medical Oncology, Dr. Ziauddin Hospital, Karachi 74700, Pakistan

**Keywords:** gastric cancer, gastroesophageal cancer, pathological response, neoadjuvant, chemotherapy

## Abstract

**Background and aim:**

Docetaxel, oxaliplatin, leucovorin and 5-fluorouracil (FLOT) may improve overall survival (OS) in patients with locally advanced gastric and gastroesophageal cancer. Our study aims to determine the pathological response in these patients with the FLOT chemotherapy in the Neoadjuvant setting. This is the first study conducted in our country.

**Methods:**

We conducted a retrospective cross-sectional study from March 2018 to December 2020. After ethical review committee approval, all patients who fulfilled the inclusion criteria and received treatment at our tertiary care center were included in the study. SPSS version 22 was used for data analysis. Frequencies and percentages were calculated for categorical. Values were presented as mean ± standard deviation (SD) for continuous variables. The chi-square test was used to determine the difference between categorical variables. A *p*-value of ≤0.05 was considered the level of significance. Kaplan-Meier curves were used to calculate survival analysis.

**Results:**

Out of 41, 35 patients with locally advanced resectable gastric or gastroesophageal adenocarcinoma were included in our study analysis. The entire cohort had a male predominance, with a mean age of 59. All patients received neoadjuvant FLOT. Pathological treatment response achieved was 77%, of which 66% had partial and 11% had complete response. There is a significant association of pathological response with age, gender, stage, grade, co-morbid and number of chemotherapy cycles received (*p*-value =<0.05). The OS was 80% with the mean OS was 2.6 years (31 months).

**Conclusion:**

Our study shows comparable response rates to other studies conducted internationally. Our findings confirm that FLOT is an effective and well-tolerated perioperative regimen with reasonable response rates in the Pakistani population. A more extensive longitudinal study would ensure these preliminary results in the local patient population.

## Introduction

Gastric and esophageal adenocarcinomas are some of the leading causes of cancer-related deaths worldwide [1]. Esophagogastric adenocarcinoma includes adenocarcinomas of the stomach (gastric cancer), the distal oesophagus and the gastroesophageal junction (GEJ) [2]. The incidence of gastric cancer in the past couple of years has been declining over time, while gastroesophageal cancer has been on a rising trend [3]. Gastric cancer, as per the literature, is the fifth most common cause of cancer around the globe and the third most common cause of cancer-related deaths, with 951,600 new cases globally recorded in 2012 [2]. In Pakistan, gastric cancers ranked seventh as per the GLOBOCAN 2020 data with a 5-year prevalence of 4.00 per 100,000 [4].

The increase in survival and decrease in mortality of gastric cancer can be attributed to a combination of early detection, better access to care and improved treatment options available [5]. Another important factor is a change in lifestyle and environmental exposures over succeeding generations. The increase in the incidence of gastroesophageal cancer can be attributed to the rise in the prevalence of gastroesophageal reflux disease, Barrett's oesophagus, abdominal obesity and reduced intake of fruits, vegetables and low-fibre diet, among many causes [6]. Symptoms include weight loss, dysphagia, dyspepsia, vomiting, early satiety, or iron deficiency anaemia. Surgical resection is by far the only curative option available for early-stage disease without suspected lymph nodes. Locally advanced and node-positive diseases should be treated via a multi-disciplinary approach [7]. Similarly, early-stage esophageal cancer can be treated with chemoradiation alone. Despite significant advances in the multimodal approach to locally advanced esophageal and esophageal junction cancer, the 5-year survival of patients treated with curative intent remains poor, in between 30% and 47% in the recent series [8].

Neoadjuvant treatment, either with chemotherapy or a combination of chemotherapy with radiation followed by surgery and adjuvant treatment, is now the current standard of care, rather than surgery alone, based on randomised clinical trials [9]. Neoadjuvant treatment aims to increase resection rates, including complete resection, decrease local and systemic recurrence and increase overall survival (OS) rates [9]. The survival benefit of perioperative treatment in gastric and GEJ cancer has also been demonstrated in clinical trials [10].

The need for a balance between a neoadjuvant treatment and dealing with acceptable toxicities remains a significant challenge for patients with resectable gastric and gastroesophageal cancers. We aim to investigate pathological response rates after neoadjuvant systemic therapy in our population. As per our knowledge, no similar study has been conducted in this part of the world. Our analysis is of utmost importance in providing valuable data regarding treatment with the neoadjuvant docetaxel, oxaliplatin, leucovorin and 5-fluorouracil (FLOT) regimen, which is easier to administer and offers better response rates than the conventional treatment options.

## Methods

This retrospective study was conducted in the Department of Medical Oncology at a large tertiary care hospital in Karachi, Pakistan, from March 2018 to December 2020. It was a descriptive observational study. Forty-one patients with biopsy-proven locally advanced gastric or gastroesophageal adenocarcinoma who were being treated with curative intent were included, however, six patients were lost to follow-up after the initial presentation, and no complete data was available. A total of 35 patients were therefore included in our study analysis. The patients included in the study were between the age group of 18–75 years, with a mean age of 59.8 ± 9.9, with biopsy-proven locally advanced resectable gastric or gastroesophageal adenocarcinoma. The study did not include patients who had distant metastasis, peripheral neuropathy grade II, cardiac insufficiency, Eastern Cooperative Oncology Group (ECOG) 3-4, uncontrolled medical illness, acute infection, or history of other malignancies within the past 5 years.

### Data collection and treatment

ERC approval was obtained, and all the patients who fulfilled the eligibility criteria were included in the study. All the patients were being treated per the standard of care and at the primary physician's discretion. No intervention was performed as part of this study. Patient demographic data was collected along with clinical outcomes. Baseline laboratories were checked before administration of chemotherapy, and if they were fine, chemotherapy was proceeded with. They received a preoperative systemic chemotherapy regimen with Docetaxel 50 mg/m^2^, Oxaliplatin 85 mg/m^2^, leucovorin 200 mg/m^2^, all on day 1 and 5-FU 2,600 mg/m^2^ as a 24-hour infusion on a 2-weekly interval. The response was evaluated after completion of neoadjuvant treatment with a computerised tomography scan of the chest, abdomen and pelvis or positron emission tomography scan. Then, the pathological stage was determined via the histological outcome after surgery. Patients received adjuvant FLOT 4–6 months after surgery and were on close follow-up with history and physical examination every 3–6 months for 2 years and then 6–12 months after that, along with a CT scan every 6 months for 2 years and then annually. The patients were being followed up for a duration of 4 years.

### Dose modification for toxicities

In the study cohort, eight (*n* = 8, 22.8%) patients needed dose adjustments in the regimen (Table 1). Out of which, two patients needed dose adjustment of the entire regimen secondary to grade III neutropenia, grade II neuropathy and grade III diarrhoea. Two patients needed dose adjustment in docetaxel infusion secondary to fatigue and grade II neuropathy. Two patients needed dose adjustment in 5-FU secondary to grade III diarrhoea and severe (Grade III-IV) oral mucositis. Two patients had to be switched to another regimen in the last cycle secondary to a decline in performance status and poor overall tolerance to the regimen. Furthermore, the majority (97%) of patients received at least three cycles of neoadjuvant FLOT chemotherapy, and 77% of patients received at least three cycles of FLOT in the adjuvant setting. Four patients received no adjuvant treatment. Four patients received another chemotherapy regimen including oral capecitabine or FOLFOX in the adjuvant setting.

### Statistical analysis

SPSS version 22 was used for data analysis. Frequencies and percentages were calculated for categorical variables such as gender, ECOG status, co-morbid (DM/Hypertension/IHD/CVA), pre-treatment clinical stage (stage I–IV), dose modifications in neoadjuvant regimen, the reason for dose modification of neoadjuvant regimen (no response/suboptimal response, disease progression/ intolerance), surgery performed (yes/no), pathological stage (stage I–IV), pathological grade (grade-I–III) and pathological response (complete/partial). Values were presented as mean ± SD for continuous variables like age and number of chemotherapy cycles received. The chi-square test was used to determine the difference between categorical variables. A *p*-value of ≤0.05 was considered the level of significance. Kaplan-Meier curves were used to calculate survival analysis.

## Results

A total of 35 patients with locally advanced resectable gastric or gastroesophageal adenocarcinoma were included in our study. The mean age was 59.875 ± 9.908 years. The descriptive statistic of age is presented in Table 1. Most patients (*n* = 33, 94.3%) were males and had an ECOG status of 1 in 33 (94.3%), as shown in Table 1. The pre-treatment clinical stage was stage 1 in 1 patient (2.9%), stage II in 10 (28.6%), stage III in 24 (68.5%). Histological subtypes include gastric adenocarcinoma (*n* = 30, 85%) and GEJ adenocarcinoma (*n* = 5, 15%). Among these, further histological sub-types consist of; well differentiated (*n* = 5, 14.3%), moderately differentiated (*n* = 12, 34.3%), poorly differentiated (*n* = 11, 31.4%), poorly differentiated with signet ring cell morphology (*n* = 6, 17.1%) and mucinous adenocarcinoma (*n* = 1, 2.9%). The median number of chemotherapy cycles received was 4 (IQR, 1–6). Baseline characteristics are shown in Table 1.

**Figure 1.**
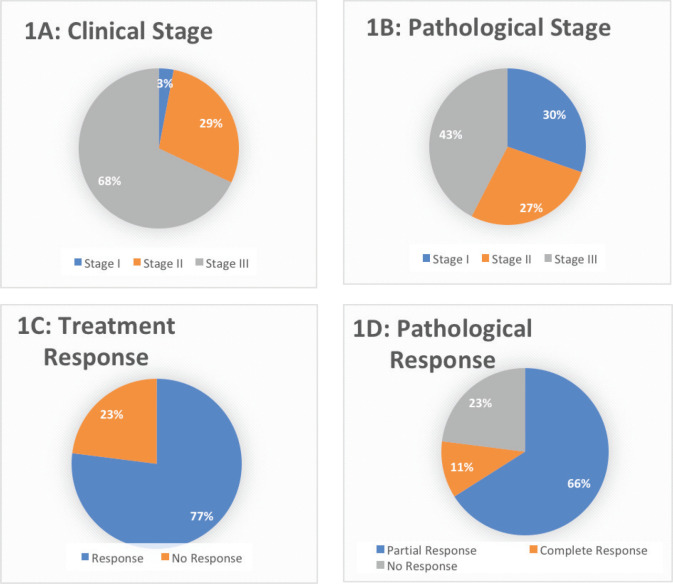
Clinical (1A) and pathological (1B) stage and the overall treatment response (1C-D) in the entire cohort.

The surgery was performed on 33 (94.28%) patients (Table 1). In the study cohort, majority (51.42%) of the patients underwent total gastrectomy + D2 resection. Partial gastrectomy + D2 resection was performed in 22.8% of the patients. Two-staged esophagectomy and distal gastrectomy were performed in 14.28% and 2.8% of the patients, respectively. Surgery was not done for two patients as one patient was not medically fit for surgery because of a decline in performance status and the other patient developed metastatic disease on scans post neoadjuvant chemotherapy. Moreover, incomplete surgery was performed in one as the patient was found to have metastatic disease with peritoneal deposits during surgery. The pathological stage was stage I in 10 (30.30%) patients, stage II in 9 (27.27%) patients and stage III in 14 (42.42) patients, as shown in Table 2 The pathological grade was grade I in 7 (20%), grade II in 11 (31.4%) and grade III in 17 (48.6%). The pathological response achieved was 77% (*n* = 27), out of which complete pathological response (PCR) was observed in 4 (11.4%) and partial response was seen in 23 (66.7%), as shown in Table 2 (Figure 1a–d).

In our study, there is a significant association of pathological response with age, gender, stage, grade, co-morbid and number of chemotherapy cycles received (Table 3).

**Figure 2.**
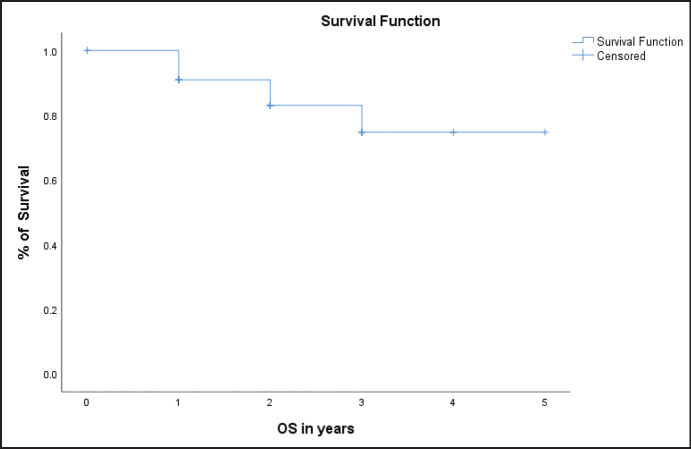
Overall survival (OS) in the entire population.

In our study, the OS for 2 years was 80% with a mean OS of 2.6 years (31 months) (Figure 2). Among the cohort of 35 patients, 6 patients experienced mortality. One patient died within the first year following the initiation of FLOT therapy due to cardiopulmonary arrest. Another patient after the first year due to complicated pneumonia, and the remaining four patients at 2, 3 and 4 years, respectively, after initiation of the therapeutic intervention.

## Discussion

The incidence of gastric cancer has been declining; however, it remains a major cause of cancer death, globally. Attempts to enhance treatment outcomes beyond those achieved through surgery alone have involved both adjuvant (postoperative) and neoadjuvant (preoperative) strategies. Over time, the beneficial effects of neoadjuvant chemotherapy on the survival of individuals with locally advanced gastric adenocarcinoma have become increasingly evident. Nevertheless, there remains a lack of consensus regarding the optimal approach. Determining the most effective chemotherapy regimen for neoadjuvant therapy remains elusive, with practice varying considerably. While the FLOT regimen has shown superior 5-year survival and disease-free survival (DFS) rates compared to previous treatments for gastric cancer and GEJ cancer [11–13], it remains unclear whether similar outcomes can be achieved in our patient population.

In our study, the overall pathological response rate after neoadjuvant FLOT in Gastric cancer (GC) and GEJ cancer was 77%. The CR was achieved in 11.4% of patients, and a partial response was seen in 66.7%. Our results are similar to most of the studies published in the literature [11–15]. Moreover, in the published literature, as compared to the Neo-FLOT study, PCR with the FLOT regimen reached 20%, and partial response reached 40% [15, 3].

The role of radiation therapy concurrent with chemotherapy in GC and GEJ cancer has been extensively investigated in multiple clinical trials. Perioperative chemoradiation is associated with improved DFS, OS and PCR compared with chemotherapy or surgery alone in early-stage cancer. Results from the multicenter phase 3 CROSS trial showed that perioperative chemoradiation with carboplatin and paclitaxel significantly improved DFS and OS with surgery alone in patients with resectable GEJ cancer. The R0 resection rate was higher in the perioperative chemoradiation arm compared to the surgery alone arm (92% versus 69%;* p* < 0.001). Median OS was 49 months in the chemoradiation arm versus 24 months in the surgery-alone arm [8].

The survival benefit of perioperative chemotherapy in GEJ cancer was first demonstrated in a landmark phase 3 MAGIC trial. This study, which compared perioperative epirubicin, cisplatin and fluorouracil (ECF) to surgery alone, established that perioperative ECF improves DFS and OS [16–18]. However, in the FLOT4 trial, PCR reached 15% and 16% in gastric and GEJ cancers, respectively. The FLOT4 study compared the FLOT regimen that is Docetaxel 50 mg/m^2^, Oxaliplatin 85 mg/m^2^, leucovorin 200 mg/m^2^ all on day 1 and 5-FU 2,600 mg/m^2^ as a 24-hour infusion four cycles preoperatively followed by surgery then four cycles postoperatively to ECX/ECF three cycles preoperatively followed by three cycles after surgery showed that the median progression-free survival was 30 months in the FLOT group versus 18 months in the ECF arm. The OS was also better in the FLOT arm, which is 50 months versus 35 months [17–19]. Our study also showed a significant survival benefit of neoadjuvant FLOT, with mean OS reaching 31 months. In our study, moreover, because of the small sample size as well as possible differences in the protoplasm/genetic variation, might be one of the reasons apart from treatment adherence and completion of treatment that leads to the relatively lower survival rate. A propensity score-matched retrospective study from China also suggested that the patients with neoadjuvant FLOT had improved OS compared with surgery first. The results of these studies indicated that the FLOT was beneficial to locally advanced gastric cancer (LAGC) in terms of pathological regression and survival [13, 15, 3, 16–19]. Hence, perioperative systemic treatment is now the standard of care for resectable gastric and gastroesophageal cancer.

In the era of immunotherapy and targeted therapy, progress has been made in personalised treatment for advanced gastric and esophagogastric cancers. The current revised 2010 histologic classification of gastric cancer by the World Health Organisation in 2019 did not consider the molecular profiling of gastric cancer [20]. The cancer genome atlas (TCGA) proposed a classification based on molecular profiling into four sub-groups, namely, Epstein bar virus tumours (9%), microsatellite instability (MSI) tumours (21%), gnomically stable tumours (20%) and chromosomal instability tumours (50%) [21]. Following this, the Asian Cancer Research Group conducted a study built on TCGA molecular classification and co-relate it with clinical outcomes and identified four distinct subtypes, including MSI-High, Microsatellite stability (MSS)/epithelial-mesenchymal transition, MSS/TP53 intact and MSS/TP53 loss [22]. These molecular mechanics have a significant role in identifying novel targeted treatments. Currently, Her-2, also known as ERBB2, and combined positive score/tumour proportion score have been identified as therapy targets in advanced-stage disease via Trastuzumab and Immunotherapy including Pembrolizumab, Nivolumab and Camrelizumab, respectively [23–27]. Nevertheless, exciting immunotherapy results in neoadjuvant settings from phase II data, which evaluates Nivolumab and Ipilimumab in dMMR gastric and GEJ cancer, have shown that 59% of patients had PCR [28]. A phase II study also explored spartalizumab in combination with FLOT in a GASPAR trial as a perioperative treatment for gastric and GEJ adenocarcinoma to improve treatment efficacy and overall outcomes [14]. Additional follow-up studies and randomised trials are required to prove this claim.

Although with certain limitations of a single institutional study and a smaller sample size, our study demonstrated that preoperative FLOT regimen chemotherapy responds well in patients with LAGC. The use of the FLOT regimen as neoadjuvant chemotherapy should be taken into consideration during the comprehensive treatment of patients with LAGC. Rigorous randomised studies are needed to determine the role of FLOT and optimal patient selection in the Pakistani population.

## Conclusion

Our study underscores the favourable impact of neoadjuvant FLOT regimen chemotherapy on locally advanced gastric and esophagogastric cancer within our local population. Notably, we observed a substantial overall pathological response rate of 77%, with a partial response recorded in 66% of cases. These findings align cohesively with existing literature, reinforcing the efficacy of the FLOT regimen and its potential to yield promising outcomes. Furthermore, our results contribute significantly to the expanding body of evidence emphasising the survival advantages associated with perioperative systemic treatments, particularly FLOT, in resectable gastric and gastroesophageal cancer. We advocate that all eligible patients stand to benefit from this approach. As we transition into an era marked by molecular and targeted treatments, ongoing trials exploring immunotherapy in the neoadjuvant setting exhibit promising signs regarding pathological response. However, a more comprehensive dataset, particularly concerning OS benefits, is crucial before considering a shift in the standard of care.

## List of abbreviations

CPS, Combined positive score; DFS, Disease-free survival; ECOG, Eastern Cooperative Oncology Group; FLOT, 5 FU, leucovorin, oxaliplatin and docetaxel; GEJ, Gastroesophageal junction; LAGC, Locally advanced gastric cancer; OS, Overall survival; PCR, Pathological complete response and PET-CT, Positron emission tomography.

## Conflicts of interest

All the authors declare no competing interest.

## Funding

The author(s) received no funding for the research and/or publication of this article.

## Ethical approval

The Aga Khan University Hospital Ethical Review Committee (AKUH-ERC) approved our study.

## Data availability

The data that has been used is confidential.

## Author contributions

TD: Conceptualisation, writing, perform the experiment, contributed reagents, analysed and interpret the data.

YR: Conceptualisation. Perform the experiment.

SRK: Writing, analysed and interpret the data.

AJ, NZ, MM: Conceived and designed the experiments.

## Figures and Tables

**Table 1.** Baseline characteristics of participants receiving FLOT regimen in the neoadjuvant setting for esophagogastric and gastric cancer.

Characteristics	*n* (%) *n* = 35
Age Mean ± SD	59.8 ± 9.9
Sex Male Female	33 (94.3) 2 (5.7)
ECOG status 1 2 3 4 0	33 (94.3) 2 (5.7) 0 (0.0) 0 (0.0) 0 (0.0)
Co-morbidities None Diabetes mellitus Hypertension Hepatitis B/C Others	18 (51.5) 6 (17.1) 6 (17.1) 2 (5.7) 3 (8.6)
Pre-treatment clinical stage Stage I Stage II Stage III	1 (2.9) 10 (28.6) 24 (68.5)
Neoadjuvant chemotherapy number of cycles Median, IQR	4 (1.6)
Dose modification No dose modification Dose modification performed.	27 (77.2) 8 (22.8)
Reason for dose modification (*n* = 8) Grade III neutropenia + Grade II–III diarrhoea + Grade II neuropathy Grade III neuropathy and fatigue Grade III-IV oral mucositis + Grade III diarrhoea Poor tolerance and decline in ECOG	2 (5.7) 2 (5.7) 2 (5.7) 2 (5.7)
Surgery performed No Yes	2 (5.7) 33 (94.3)
Type of surgery (*n* = 33) Total gastrectomy + D2 resection Partial gastrectomy + D2 resection Distal gastrectomy Two staged esophagectomy Incomplete surgery	18 (51.42) 8 (22.8) 1 (2.8) 5 (14.28) 1 (2.8)

**Table 2.** Disease characteristics and response rates.

Disease characteristics	*n* (%)
Pathological stage Stage I Stage II Stage III	10 (30.33) 9 (27.27) 14 (42.42)
Pathological grade Grade I Grade II Grade III	7 (20.0) 11 (31.4) 17 (48.6)
Treatment response Response achieved No response	27 (77.1) 8 (22.9)
Pathological response rate Partial response Complete response	23 (66.7) 4 (11.4)

**Table 3.** Pathological response stratified based on patients and disease characteristics.

Characteristics	Pathological response			*p*-value
	No response (*n* = 8)	Partial (*n* = 23)	Complete (*n* = 4)	
Age Mean ± SD	57.2 ± 10.6	60.9 ± 10.3	58.7 ± 5.5	0.001
Sex Male Female	8 (100.0) 0 (0.0)	22 (95.7) 1 (4.3)	3 (75.0) 1 (25.0)	0.05
ECOG status 1 2 3 4 0	8 (100.0) 0 (0.0) 0 (0.0) 0 (0.0) 0 (0.0)	21 (91.3) 2 (8.7) 0 (0.0) 0 (0.0) 0 (0.0)	4 (100.0) 0 (0.0) 0 (0.0) 0 (0.0) 0 (0.0)	0.73
Co-morbidities None DM Hypertension Hepatitis B/C Others	6 (75.0) 0 (0.0) 2 (25.0) 0 (0.0) 0 (0.0)	11 (47.8) 5 (21.7) 2 (8.7) 2 (8.7) 3 (13.1)	1 (25.0) 1 (25.0) 2 (50.0) 0 (0.0) 0 (0.0)	0.02
Pre-treatment clinical stage Stage I Stage II Stage III	1 (12.5) 0 (0.0) 7 (87.5)	0 (0.0) 7 (30.4) 16 (69.6)	0 (0.0) 3 (75.0) 1 (25.0)	0.01
No. of chemo cycles Median	4	4	4	0.001
Pathological stage Stage I Stage II Stage III	0 (0.0) 0 (0.0) 6 (75.0)	8 (34.8) 8 (34.8) 7 (30.4)	2 (50.0) 1 (25.0) 1 (25.0)	0.02
Pathological grade Grade I Grade II Grade III	1 (12.5) 1 (12.5) 6 (75.0)	5 (21.8) 9 (39.1) 9 (39.1)	1 (25.0) 1 (25.0) 2 (50.0)	0.05
